# Genetics and genomic medicine in Argentina

**DOI:** 10.1002/mgg3.455

**Published:** 2018-07-26

**Authors:** Sebastián A. Vishnopolska, Adrián G. Turjanski, Mariana Herrera Piñero, Boris Groisman, Rosa Liascovich, Ana Chiesa, Marcelo A. Marti

**Affiliations:** ^1^ Departamento de Química Biológica Facultad de Ciencias Exactas y Naturales Universidad de Buenos Aires Buenos Aires Argentina; ^2^ Instituto de Química Biológica de la Facultad de Ciencias Exactas y Naturales (IQUIBICEN) CONICET Universidad de Buenos Aires Buenos Aires Argentina; ^3^ Banco Nacional de Datos Genéticos (BNDG) Ministerio de Ciencia Tecnología e Innovación Productiva Buenos Aires Argentina; ^4^ Red Nacional de Anomalías Congénitas (RENAC) Centro Nacional de Genética Médica (ANLIS) Ministerio de Salud Buenos Aires Argentina; ^5^ Fundación de Endocrinología Infantil División de Endocrinología Hospital de Niños Ricardo Gutiérrez Centro de Investigaciones Endocrinológicas Dr. César Bergada (CEDIE) Buenos Aires Argentina

## Abstract

A historical summary of genetics and genomic medicine in Argentina. We go through the achievements and difficulties in the implementation of genetic and genomic services both in academia and health care.

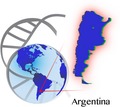

## 1 ARGENTINEAN DEMOGRAPHIC FEATURES

**Figure 1:**
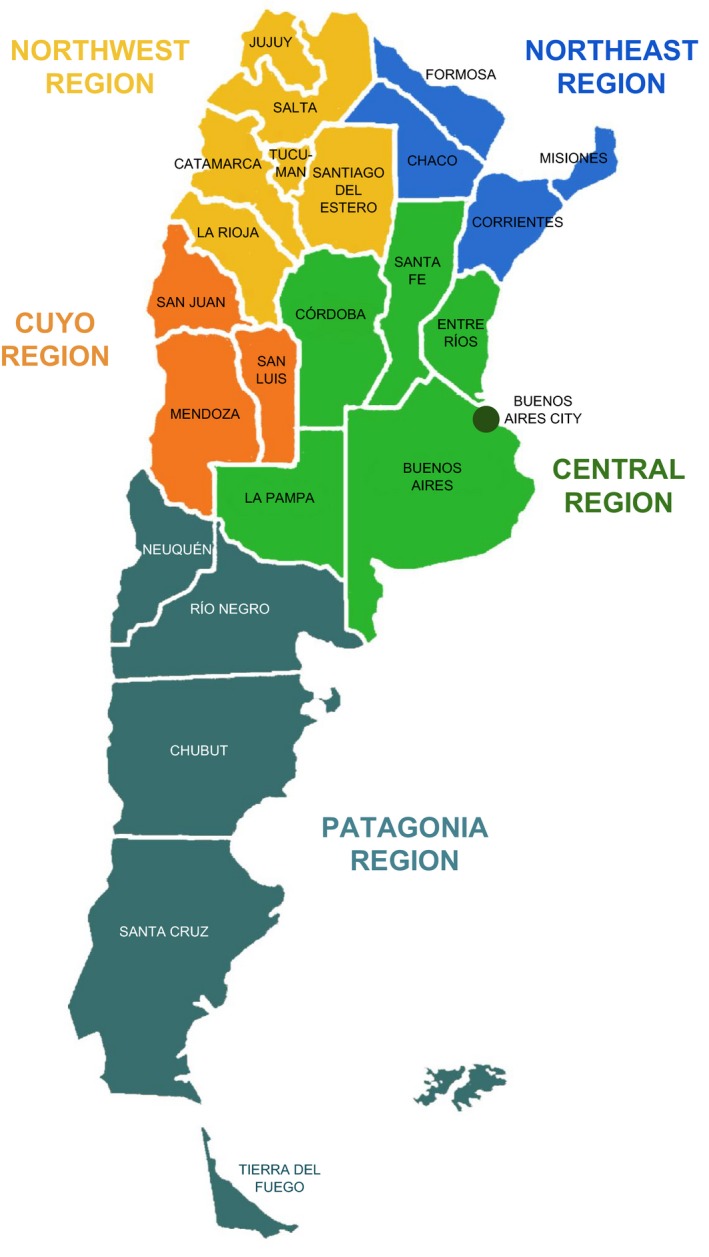
Economic regions and provinces of Argentina

Located in the southernmost region of Latin America, Argentina is the largest Spanish‐speaking country in the world with an estimated area of 2,791,810 sq. km of continental land (plus 969,464 sq. km of islands and Antarctic land). It is bordered on the west by Chile, on the north by Bolivia and Paraguay, and on the east by Brazil, Uruguay, and the Atlantic Ocean. From a historical and geographical viewpoint, Argentina can be divided into five regions: central (the most populous), west (Cuyo), northwest, northeast, and south (Patagonia) (Figure 1). The continental area is divided into 23 provinces plus the capital city, Buenos Aires, harboring an estimated total population of 44 million people (INDEC, 2010; United Nations Data, 2017), living 91% in urban areas. Around 65% of the population is concentrated in the central region provinces, particularly in the province of Buenos Aires, with 38.95% of the country population.

Argentina's population was heavily influenced my numerous migration waves, mostly from Europe, that admixed with the original Amerindian communities. Several studies (Avena et al., 2012; Homburger et al., 2015; Seldin et al., 2007) concluded that the ethnical composition of Argentina is mostly composed by European descent (52%–78%), followed by Amerindian (19%–31%), African (2%–9%), and Asiatic (1%). Although the European genetic imprint is high, it is estimated that most inhabitants do have some Amerindian admixture. (Avena et al., 2012). Additionally, in Argentina inhabit, there were several ethnic minorities such as German, Arab, Polish, Jewish, Armenian, Peruvian, Chilean, Uruguayan, Japanese, Chinese, and Korean.

## 2 HEALTH CARE IN ARGENTINA

The healthcare system in Argentina is divided into three settings: public, social security, and private insurance. The public system is funded through taxes and is available free of charge to the entire population. It handles approximately 46% of the population, mainly those of lower income, who lack other health coverage. The social security setting is comprised of labor union‐based coverage, funded by mandatory contributions from employers and registered workers, covering about 44% of the population (workers, employees, and retirees). The private insurance setting (for‐profit) is funded by specific payments from the insured and serves 10% of the population, mainly the higher income fraction. The three systems are independently managed with little interaction between them, which results in overlapping, inefficiency, and high health expenditure (about 6.61% of the Gross Domestic Product). (DEIS, 2010).

The National Ministry of Health has a leading role in public health policy, being responsible for introducing specific regulations, and running national health programs (i.e., immunizations, noncommunicable diseases, sexual and reproductive health and maternal and child health). Provincial ministries of health are autonomous in terms of planning and decision‐making on human resources, purchases, infrastructure, and other actions to provide services to their people. Given its different settings and administrative jurisdictions, the Argentine healthcare system can be considered a fragmented model (PNUD, 2011).

There are more than 25 thousand different health centers countrywide. The combined life expectancy at birth is 76.7 years, which is the third greatest in the region (80.4 years for women and 73.0 for men) (PAHO/WHO, 2017). The natality and adult mortality rate are 17.9 and 7.7 for every 1000 habitants, and the child death rate is 9.7 per 1,000 births (DEIS, 2015b). In 2015, 99.5% of births occurred in official health centers, highlighting a high degree of institutionalized deliveries (Langou & Sachetti, 2017). Although the child mortality rate has been going down steadily in the last decades, mainly due to improvement in prevention and treatment of infectious diseases and pre‐ and postnatal nutritional factors, the value is still high, with congenital defects a major cause (ca. 26%) (DEIS, 2015a).

## 3 EARLY GENETIC TESTS IN ARGENTINA: GENETICS APPLIED TO FORENSIC STUDIES

Between years 1976 and 1983, Argentina went through one of the cruelest and bloodiest military dictatorships of Latin America. About 30,000 people were kidnapped, tortured and killed, and their bodies remain missing. In Argentina they are called “Desaparecidos” as disappeared or vanished by the de facto government. (CONADEP, 1984). Argentina's history of genetics is tightly related to forensic development and applications that contributed to solve identity crimes performed in this dark period.

Many women undergoing pregnancies or kidnapped along their children were taken to clandestine detention centers, where they were tortured, gave birth in subhuman conditions, separated from their offspring, and ultimately shot or pushed from airplanes to drown in the Río de la Plata river—what were called “flights of death”. About 500 kidnapped children were never restored to their biological families. They were abandoned in the streets, taken to foster homes or given to foster families of militaries (or friends of them), erasing their identities and depriving them of their true biological links and history. This repeated practice evidences that, in Argentina, there was a systematic plan of appropriation and stealing of babies by the Armed Forces (Nosiglia, 1985).

Year 1984 marked an inflection point in the history of Science and Human Rights, both in Argentina and the world. Democracy was again restored, and the grandmothers of missing children—joined in the “Asociación Abuelas de Plaza de Mayo” (Grandmothers of Plaza de Mayo Association)—were visited by a delegation of the American Association for the Advancement of Science (AASS) in order to develop a scientific method that would allow the identification of the robbed grandchildren, especially in the absence of both parents due to forced disappearance. The method was based in the study of the Histocompatibility Antigens and Blood Groups inheritance pattern and led to the development of the first statistical value for the “index of grandpaternity” (Di Lonardo, Darlu, Baur, Orrego, & King, 1984). In December of 1984, an 8‐year‐old girl named Paula Eva Logares Grinspon, previously kidnapped along her parents when she was 2 years old and then appropriated by a member of the police forces, was returned to her biological family with the help of these genetic studies.

In 1987 by National Law 23,511, it was the National Bank of Genetic Data (BNDG) was created, whose aim is to collect the genetic profiles of all relatives of missing grandchildren in order to compare them with potential “kidnapped” children. This bank was the seed and inspiration to many other genetic banks that sprouted in the early nineties in order to identify missing people, victims of human trafficking, and stealing of children (ICMP 2018).

Different and new forensic genetic techniques were employed and added during the following years. Initially histocompatibility and maternal lineage studies using mitochondrial DNA sequencing enabled the recovery of grandchildren in Argentina (Orrego & King, 1990) Later, polymerase chain reaction (PCR) also contributed to the development of new genetic markers, both in autosomal and sex chromosomes. (Ruitberg, Reeder, & Butler, 2001). Thirty years after the creation of the BNDG, new mathematical advancements were applied to the identification of missing people (Kling, Egeland, Piñero, & Vigeland, 2017) as well as new software specialized in massive search for Missing People Identification (MPI) and Disaster Victim Identification (DVI) (Brenner, 2006; CODIS 1990; Egeland, Mostad, Mevåg, & Stenersen, 2000; Kling, Tillmar, & Egeland, 2014). Table 1 shows a summary of methods employed along the years.

**Table 1:** Evolution and incorporation of forensic genetics techniques during the 30 years of the National Bank of Genetic Data (BNDG)

Stage	Time frame	Methods
Exploratory	1984–1992	HLA, RFLP, and early PCR
Stabilization	1992–2005	Mitochondrial DNA sequencing, autosomal and chromosome Y microsatellite standardization and quality controls
Growth	2005–2018	Chromosome X Microsatellites. Automatization. Software for massive searchs (DVI, MPI)

Note

DVI: disaster victim identification; HLA: human leukocyte antigen; MPI: missing people identification; PCR: polymerase chain reaction; RFLP: restriction fragment length polymorphism.

**Figure 2:**
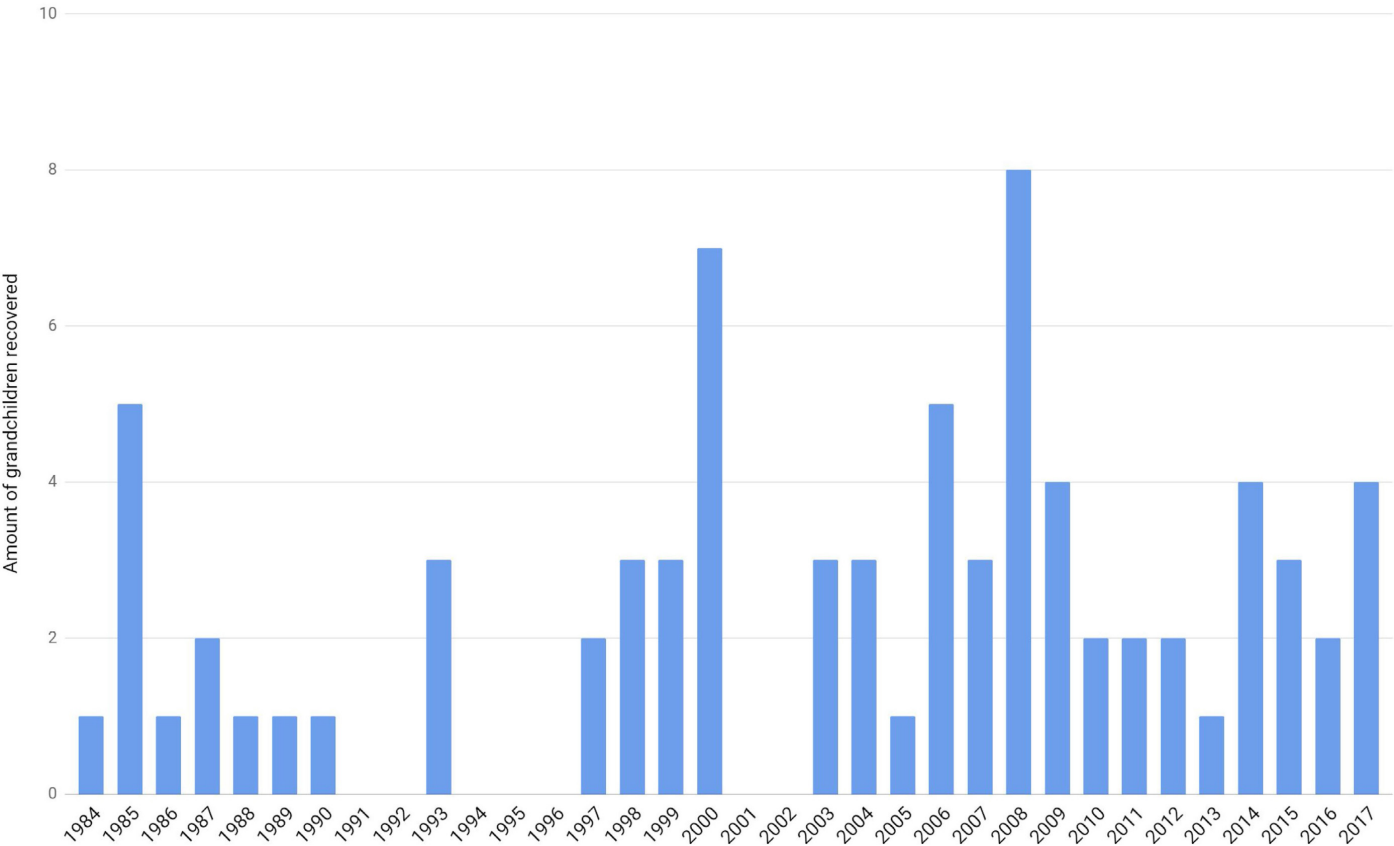
Grandchildren restored to their families by year by DNA analysis performed in the National Bank of Genetic Data (BNDG)

Up to date, 127 identifications have been achieved, 50 of them were completed without need of genetic testing, (34 children had been already born when kidnapped and directly recognized by their families, while other 16 have never been born as their mothers were found dead and buried as “No Name” and the burial dates were incompatible to them giving birth before being killed). Figure 2 shows the number of genetic‐based grandchildren recoveries performed by the BNDG over the years. Posterior analysis of the documentation of the 111 living recoveries demonstrated a behavior pattern in the appropriation felonies. About 70% of the children were registered as biological with certificates of domiciliary births signed by obstetricians linked to the Armed Forces or in clinics associated with baby trafficking. Additionally, 85% of these babies were appropriated by couples belonging to the Armed Forces or related to them. The results obtained by the BNDG also served as proof in the numerous trials for crimes against humanity perpetuated in our country (Data supplied by Unidad especializada para casos de apropiación de niños durante el terrorismo de Estado. https://www.fiscales.gob.ar/unidad-de-apropiacion/).

The archives of the BNDG evidence the history and evolution of forensic genetics and are unique in a way that there is no other database for the search and identification of appropriated children in the world (Lerman, 2017). The persistent fight carried by *las Abuelas de Plaza de Mayo* (The Grandmothers of Plaza de Mayo) allowed science to stand by the side of the Human Rights, to not be used as a tool for discrimination, but for the right of identity and repairment of serious violations to human kind. (Stern, 2013 March). This year 2018, *las Abuelas de Plaza de Mayo* have been nominated for the sixth time to Nobel Peace Prize for their labor in Humans Rights (Por los más de 40 años de lucha, 2018).

## 4 MEDICAL GENETICS

The first medical GENETIC services in Argentina started in the late 1960s brought by pediatricians and obstetricians trained abroad. The National Center of Medical Genetics (CENAGEM), under the National Ministry of Health, was established in 1969, followed by other genetic services located in the main pediatric hospitals. However, the development of medical genetics services in Argentina lacked a planned strategy.

In 1979, CENAGEM started a medical genetics residency, which was followed by the cytogenetics residency. In the early years of 21st century, different surveys showed that health professionals in Argentina still had little knowledge of medical genetics (Barbero et al., 2003). Ten of the 24 jurisdictions had no genetic services in the public healthcare sector, with most of the services settled in the central region of the country (Liascovich, Rozental, Barbero, Alba, & Ortiz, 2006) and no coordination between services due to dependency from multiple authorities (academic, municipal, provincial, and/or national). Some practices, such as prenatal diagnosis, were only offered in the private setting. It is important to note, that since abortion has been illegal unless the life or health of the mother is at risk, or in case of rape, (an issue which is currently under intense debate in the congress), the development of prenatal diagnosis in the public setting is stagnant (Penchaszadeh, 2013).

Promoted by the CENAGEM, the National Program of the Genetics Network was designed in 2008 through a decree issued by the National Ministry of Health (Resolution 1227/2008). This program aimed to build a network of genetic services from the public sector and train healthcare professionals in medical genetics. Since 2012, each province appointed a local healthcare professional to work with the central coordination of this National Program. The central coordination of the program trained locally selected health professionals, which led to strengthen local genetic services. Among new initiatives addressing birth defects in Argentina, there is a new area working on surveying, diagnosing, and treating rare diseases, organized after a national law on the topic was enacted (National Law 26.689, 2011). Nowadays, most genetics services make clinical and cytogenetic diagnoses and only a few perform molecular diagnostics for some specific monogenic diseases.

## 5 NEONATAL SCREENING OF GENETIC DISORDERS

Newborn screening (NBS) describes the procedures that, if performed during the first few hours or days of a newborn's life, have the potential of preventing severe health problems, including death. Traditionally based on biochemical studies, evolution of NBS has boosted in the genomic era, nowadays being able, in principle, to cover—or detect—hundreds of conditions for which early detection and treatment modify the outcome (Friedman et al., 2017). In Latin America, NBS began in the early 70s with the Cuban experience, quickly followed by Argentina and other countries. (Borrajo, 2007; Therrell & Adams, 2007; Therrell et al., 2015). Each country, depending on the health circumstances and available resources, offers a different program regarding disorders, methodology, and screening strategies.

In Argentina, the neonatal screening program for congenital hypothyroidism (CH) and phenylketonuria (PKU) carried out by “Fundación de Endocrinología Infantil” (FEI)—a nonprofit institution closely related to the Division of Endocrinology Research in the Buenos Aires Children's Hospital R. Gutiérrez started in August 1985 with the systematic detection of these diseases in several maternities along the country (Chiesa et al., 2013). Initially, screening was not mandatory by law; thus, it was implemented by “request.” The slogan “a single drop of blood can prevent a sea of tears” aimed to call the attention of parents and doctors about the need of testing every baby. As awareness of doctors and health authorities grew, screening practices began to increase, and FEI became the referent organization for guidelines for National NBS development.

In 1986, national law (Law Nr. 23,413) obliged to test every newborn for PKU. CH and Cystic Fibrosis (CF) were added also by law in 1990 and 1994, respectively. Finally, with the approval of a new law in 2007 (Law Nr. 26,279), it was stated that every newborn in Argentina had to be tested to detect and treat PKU, CH, CF, galactosemia, congenital adrenal hyperplasia, biotinidase deficiency, preterm retinopathy, Chagas disease, and syphilis. Moreover, the law stated that other diseases (genetic, metabolic, or congenital) inapparent at birth should be screened if there was a medical justification and/or political health needs (MSAL 2011).

The first public funding program (PRODYTEC) for NBS was held in 1995 by the Argentine Biochemical Foundation (FABA) in Buenos Aires province and in 2001, Buenos Aires' city government started its own program giving full coverage to babies born in the city's public maternity hospitals. In the national arena, the “National Program for Neonatal Screening Strengthening” was held in 2006 with the aim of helping those provinces without NBS programs or with them but experiencing difficulties. Although each province had its own NBS laboratory, the experience was centrally coordinated in Buenos Aires.

Following international guidelines, other diseases were consecutively included in many of these programs. Nowadays, screening for all previous stated diseases are performed routinely in all programs, while Maple Syrup Urine Disease (MSUD) was included in the Buenos Aires Province, FEI, and the Buenos Aires City programs. Since 2014, with availability of the MS/MS methodology, the Buenos Aires City program began with Medium‐chain acyl‐CoA dehydrogenase (MCAD) deficiency screening.

Although the estimated coverage of NBS in Argentina surpasses 90% of newborns, it is important to mention that: (a) not all the babies are tested for the same disorders or undergo the same procedures and (b) not all studies necessarily follow a precise molecular genetic diagnostic test (i.e., identification of mutation responsible for disease development).

Table 2 shows the number of newborns screened and the mean estimated incidence of each disorder since the start of the FEI NBS screening program (1985), the Biochemical Foundation screening program (1995), the Buenos Aires City screening program (2001), and the National program for the strengthen of neonatal screening (2006), up to December 2017 (kindly provided by each program). The disease marker, methodology, and cutoff chosen for detection may have varied between programs and along the years, but the data included assure the correct interpretation and confirmation of every case. As shown, primary CH, screened by TSH levels, is the most prevalent condition in the population. Congenital adrenal hyperplasia—mainly the salt wasting forms—is detected by the neonatal rise in 17 OH‐progesterone levels, evidencing the enzymatic blockade. The search for hyperphenylalaninemia is based on the determination of phenylalanine levels or on the phenylalanine/tyrosine ratio, when MS/MS methodology is available. The disease is more frequent in populations with European ancestors and less frequent in those regions with more native ethnicity, so dissimilarities in incidence may be found by different programs (Borrajo, 2007). CF is prevalent in our population and its screening is complex because immunoreactive trypsin (IRT) is a relatively weak marker. For that reason, a second step has to be added to the screening strategy. In our country, another IRT or pancreatitis‐associated protein (PAP) assay is preferred over molecular testing for confirmation, due to its lower cost. Total galactose allows the identification of the galactosemic state produced by any of the three enzymatic defects in the galactose pathway. Detection of Biotinidase deficiency is worth screening for in our population where total and partial forms are found. Finally, MSUD is not frequent in our population and the recently included screening for MCAD does not have enough number of screened babies to reflect its real incidence.

**Table 2:** Incidence of diseases screened referred to the number of screened newborn as reported by four NBS Argentinean programs (1985–2017) in Argentina

Disease	Disease marker	Number of screened babies up to 12/2017	Incidence
Congenital hypothyroidism	TSH	8,855,085	1:1,938
Congenital adrenal hyperplasia	17OH Progesterone	5,426,789	1:14,004
Phenylketonuria/hyperphenylalaninemia	Phenylalanine	8,891,158	1:31,087 1:31,754
Cystic fibrosis	(IRT‐IRT)/(IRT‐PAP)	5,444,430	1:8,753
Galactosemia	Total galactose	5,670,649	1:63,000
Biotinidase deficiency	Biotinidase activity	5,232,514	1:120,000
Maple syrup disease	Leucine	1,345,061	1:149,451
Medium chain AcylcoA dehydrogenase deficiency (MCAAD)	C8 C10	112,926	1:112,926

Note

C10: decanoylcarnitine; C8: octanoylcarnitine; IRT: immunoreactive trypsin; PAP: pancreatitis‐associated protein; TSH: thyroid‐stimulating hormone.

## 6 SURVEILLANCE OF BIRTH DEFECTS

Birth defects (BD), also called congenital anomalies, are structural or functional defects that originate during the prenatal period. BD are etiologically heterogeneous, either multifactorial (predisposing genes that are expressed in the presence of environmental triggers), predominantly genetic (chromosomal or monogenic abnormalities), or predominantly environmental (prenatal exposure to teratogenic agents) (Stevenson, Hall, & Goodman, 1993). Around 94% of newborns with birth defects are born in low‐ and middle‐income countries, placing an additional burden on families, communities, and health systems (Christianson, Howson, & Modell, 2006).

As previously stated, in Argentina, BD are a leading cause of infant deaths. Among 7,093 infant deaths that occurred in 2016, 2,175 (30%) were due to BD (DEIS, 2016). In 2009, members of CENAGEM started the implementation of the National Network of Congenital Anomalies of Argentina (RENAC). RENAC objectives are to monitor continuously and systematically the frequency of BD; respond to alarms linked to possible epidemics of congenital anomalies; evaluate risk factors that contribute to the occurrence of BD in Argentina and the impact of preventive population interventions; and promote the training of maternity health teams for the recognition of BD in newborns and their timely referral to care services (Groisman, Bidondo, Gili, Barbero, & Liascovich, 2013; Groisman, Bidondo, Barbero, et al., 2013).

The RENAC is an official and hospital‐based surveillance system, which includes 134 public and 26 nonpublic hospitals from the 24 provinces of the country. Annual coverage is approximately 300,000 births, which accounts for 65% from the public health sector and 38% of all births in the country (RENAC, 2017). Since 2012, the RENAC has become an active member of the International Clearinghouse for BD Surveillance and Research (ICBDSR), an international consortium of BD surveillance programs (Botto et al., 2006). To ensure proper data collection, in each participating hospital, there are two RENAC “champions.” These motivated health professionals (neonatologists, pediatricians, or nurses) are in charge of collecting information about the affected cases and sending the data to the RENAC coordination through a web‐based forum. The website allows sending data, photographs, solving operational issues, and providing quality control of data. Members of the RENAC coordination have expertise in medical genetics and epidemiology.

Surveillance systems have a big potential for early detection of infants with BD, thus allowing referral to adequate health services (Botto & Mastroiacovo, 2000). As already mentioned, many physicians are not trained in the basics of medical genetics and Argentina has few and scattered genetic services, with some provinces having no genetic service at all (Liascovich et al., 2006). In this context, RENAC has developed guidelines for the consultation of patients and the training of health professionals in medical genetics. RENAC champions can enter the web forum at any time and post messages with patient photographs, x‐rays, laboratory results, and other studies that allow discussion of the clinical case (Groisman, Bidondo, Gili, et al., 2013). RENAC coordination contributes in diagnosis, detection of associated birth defects, providing tools for basic genetic counseling, and referral to genetic services. By belonging to RENAC, local teams in maternity hospitals can receive help in the diagnosis of complex cases with rare or multiple defects, or at life risk, whose families require support and counseling. This significantly improves patients' health and their families leave the maternity wards with a presumptive diagnosis that allows a shorter diagnostic journey and a proper follow‐up.

Regarding BD prevalence in the country, the most frequent BD reported by RENAC in 2016 were down syndrome, talipes, critical congenital heart defects, neural tube defects, and cleft lip with cleft palate (Table 3).

**Table 3:** Prevalence of selected birth defects over 305,452 births, RENAC, 2016

Birth defect (ICD‐10 RCPCH)	Total cases	Prevalence per 10,000 (CI 95%)
Critical congenital heart defects^a^	353	11.56 (10.38–12.83)
Neural tube defects (Q00, Q01, Q05)	270	8.84 (7.82–9.96)
Anencephaly (Q00)	57	1.87 (1.41–2.42)
Spina bifida (Q05)	175	5.73 (4.91–6.64)
Encephalocele (Q01)	39	1.28 (0.91–1.75)
Cleft lip only (Q36, excluding Q36.1)	59	1.96 (1.50–2.53)
Cleft lip and palate (Q37)	341	11.16 (10.01–12.41)
Cleft palate only (Q35)	99	3.24 (2.63–3.95)
Hypospadias (Q54.1–Q54.3)	46	2.65 (2.11–3.30)
Limb deficiencies (Q71–Q73)	153	5.01 (4.25–5.87)
Transverse (Q71.2–Q71.30	42	1.38 (0.99–1.86)
Preaxial (Q71.31, Q72.5)	19	0.62 (0.37–0.97)
Postaxial (Q71.5; Q72.9)	10	0.33 (0.16–0.60)
Talipes (Q66)	378	12.4 (11.2–13.7)
Equinovarus (Q66.0)	180	5.89 (5.06–6.82)
Calcaneovalgus (Q66.4)	16	0.52 (0.30–0.85)
Microtia/anotia (Q16; Q17.1)	88	2.88 (2.31–3.55)
Polydactyly (Q69)	239	7.8 (6.9–8.9)
Preaxial (Q69.00;Q69.1;Q69.20)	46	1.51 (1.10–2.01)
Postaxial (Q69.02; Q69.22)	148	4.85 (4.10–5.69)
Omphalocele (Q79.2)	66	2.16 (1.67–2.75)
Gastroschisis (Q79.3)	239	7.82 (6.86–8.88)
Down syndrome (Q90)	548	17.94 (16.47–19.51)

a Q20.0, Q20.3, Q20.4, Q21.3, Q21.82, Q22.00, Q22.40, Q22.5, Q23.4, Q25.1–Q25.19, Q25.2, Q26.2, Q26.20.

The CENAGEM and national NBS and RENAC BD surveillance program provide the pillars for the application of medical genetics in Argentina as well as carrying training initiatives for healthcare professionals. They are ideally suited and expected to increasingly adopt the new genomic technologies in the upcoming years. Interestingly, and as will be shown below, entry to the genomic era was initially performed by research and academia‐based initiatives.

## 7 THE GENOMIC ERA IN ARGENTINA

With the aim of implementing and settling the basis for genomic studies in the country, in 2012, the Argentinean Genomic and Bioinformatic Platforms were created as initial 4‐year projects, with the help and financing of the National Ministry of Science and Technology (MINCyT). The main aims of these platforms were to develop and transfer genomic and bioinformatic knowledge as well as facilitating those services to public and private parties.

It was during this period that, in collaboration with the local hospital “Hospital de Agudos Ramos Mejia,” the very first three whole genomes were entirely sequenced in the country: the hospital made the link with the patients and their family and got written consent from all individuals involved, DNA was extracted and sequenced in INDEAR (Agrobiotechnology institute of Rosario), as member of the genomic sequencing platform, and bioinformatic analysis was performed in the bioinformatic platform in Buenos Aires (http://www.biargentina.com.ar). These genomes comprised three siblings, all with autism spectrum disorder, whose careful analysis finally led to the discovery of a shared de novo variant in the *SHANK3* gene (OMIM 606230, Genbank NM_033517) (Nemirovsky et al., 2015). Following this first success, it was important to continue expanding the reach of new generation sequencing technologies and genomic studies to a broader group of medical specialists and patients with rare genetic diseases and no molecular diagnosis, countrywide.

With this idea in mind, in 2016, we launched a new project whose aim was to sequence 100 exomes of patients with rare genetic disorders throughout the country. The “100 Exomes Campaign” was jointly financed and performed by the research group of Dr. Marti and Dr. Turjanski at the Department of Biological Chemistry of the Faculty of Science of the University of Buenos Aires, and Bitgenia (http://www.bitgenia.com), a local Genomics company (Bitgenia, 2016). The exomes were sequenced at no cost for the patients and their families, and we performed all downstream analysis also free of charge. Variant prioritization was performed, whenever possible, jointly with the healthcare professionals in order to train them in the interpretation of genomic data. Results were provided to the physician in charge, so they could offer proper genetic counseling to the patients involved in the project.

Throughout the campaign, the project involved 58 health professionals working in 32 different public or private institutions, and included patients with a variety of clinical diagnostics such as cardiopathies, skin disorders, familial cancer, neurodevelopmental disorders, cranial malformations, inborn errors of the metabolism, mitochondrial disorders, endocrine disorders, absorption disorders, and primary immunodeficiencies or autoimmune disorders, for naming some. Among the 100 analyzed cases, for 31, we found one or more known pathogenic variants with reported evidence of association with the initial diagnosis, while for another 27 cases, we found novel likely pathogenic variants in genes previously associated with disease. Some of these variants were then confirmed to cause disease by functional studies in collaboration with other groups (Ma et al., 2017). We consider these cases, which represent about 50% of those sequenced, as potential successful diagnostics, clearly highlighting the potential of whole exome sequence for medical genetics.

## 8 NATIONAL GENOMICS DATA SYSTEM

A parallel initiative to promote and coordinate the development of genomic technologies and data sharing, led to the creation of The National Genomics Data System of Argentina (SNDG by its name in Spanish: Sistema Nacional de Datos Genómicos, http://www.datosgenomicos.mincyt.gob.ar/) in 2015, under the Ministry of Science, Technology and Innovation (Ministerial Resolution 761/14). The SNDG emerged as a natural consequence of different initiatives—including the mentioned Bioinformatics Platform—and due to the establishment of several local bioinformatics and genomics research groups. Taking this into account, the development of a national system to organize the data generated by the different projects around the country became an imperative goal.

The main objective of the system is to establish an unified national database of genomic information, for all species of ecological, agricultural, biotechnological, and health interest, derived from research and surveys taken into place or funded by the country, warranting Argentinean researchers their availability and accessibility . Parallel objectives also include training local researchers in how to generate and analyze genomic data, generating a local interdisciplinary and cooperative community of laboratory scientists and bioinformaticians, promoting and facilitating the generation of genomic information, generating quality standards both for generation and analysis of genomic data, and promoting the development of bioinformatic tools locally. Since its creation, the SNDG incorporated more than 20 centers around the country, gave grants to more than 10 projects related to genomics, and generated the pipeline and web portal to upload and analyze genomic data.

## 9 THE PRESENT I: BOOSTING MOLECULAR DIAGNOSTICS

Nowadays genomics medicine is flooding Argentina, since most government supported initiatives from both Health and Science central agencies, private healthcare institutions and clinical diagnostic laboratories are offering genomic services, either supervised by healthcare professionals or with a direct to consumer sales model. These genomic services offer either precise molecular tests for (rare) Mendelian diseases (González‐Morón et al., 2017) ‐using both whole exome or panel‐based sequencing‐; evaluation of cancer risk ‐mainly based on BRCA1/2 genes analysis (OMIM 113705, NM_007300; OMIM 600185, NM_000059) (Israel, 2013); or Non Invasive Prenatal Testing (NIPT) (Vázquez et al., 2017) of chromosomal anomalies. Sequencing is either performed locally (currently there are between 10–20 next generation sequencers in health centers in the country) or outsourced abroad. The same is true for the analysis of raw data and/or interpretation of results in a clinical context, with several health‐ or research‐based institutions harboring clinical genomics interpretation services (or even research groups, such as our own). Prices also vary greatly, possibly due to inherent differences in the nature of the provided service. They can be as high as 2,000 USD for a complete service, including sequencing and clinical interpretation, and possibly no lower than 400 USD. Interestingly, there is no correlation between the amount of sequenced/analyzed data and the total cost (i.e, a whole exome is not necessarily more expensive than a gene panel).

The wider access to genomic services (for both providers and consumers), together with increased presence of genomic‐related content in the local media, is creating an increasing demand of genomic interpretation services in our country, which contrasts with the scarce number of trained professionals in clinical genomics and related areas. To overcome this issue, not only are specialized courses in genomics for scientists and healthcare professionals needed (such as the “School of Clinical Genomics” which is held every August at the local Department of Biological Chemistry and financed by the Centro Latinoamericano de Formación Interdisciplinaria, CELFI, http://www.celfi.gob.ar/programas/detalle?p=100) but a significant increase of Molecular Genetics training in basic physicians curricula would also be welcomed. However, the current lack of local regulation of these kind of services constitutes a big setback, a fact that not only impacts the quality of the results and ethical issues related to patients' rights but also is tightly related to people's accessibility in the context of Argentina's healthcare system. This lack of proper regulation of clinical genomic services ultimately results in an accessibility bias toward the higher income strata of society.

## 10 THE PRESENT II: ARGENTINA'S “PRECISION MEDICINE INITIATIVE”

In 2017, Argentina's Ministry of Science and Technology launched a Precision Medicine Initiative Grant whose aim was to establish the scientific know‐how and protocols required to implement “omic” technologies in the clinical practice, by performing a proof of concept of the process in a small number of patients. Three grants were awarded, including ours which targeted “Clinical Genomics of Pediatric Diseases,” one related to the creation of a National Biobank, and the third related to cancer genomics. Projects involve both healthcare institutions harboring NGS equipment and research groups from academia, thus displaying a high potential for synergic collaboration. Our project started in early 2018 and is expected to develop several targeted sequencing panels for pediatric disease groups and analyze a cohort of about 1000 patients in the following years.

This initiative shows that precision medicine and genomics are part of the political scientific agenda; however, it is difficult to guess the real impact that these projects are going to have on the overall healthcare system.

## 11 CONCLUDING REMARKS

Argentina is a country with inequalities in terms of healthcare, including medical genetics. Although all inhabitants (and immigrants) by law are granted access to health services, the fragmented system of public, social security, and private funding contributes to wide disparities in the population's effective use of key technologies, especially in the genetics and genomics area.

The use of genetic testing in the forensics field for the recovery of appropriated grandchildren and the fight for human rights seems to have had little influence on human health in Argentina. Until now, the vast majority of physicians trained in the country have little knowledge of genetics and the underlying molecular basis of human diseases.

In this context, it is important to highlight the efforts of several public organizations such as BNDG, CENAGEM, RENAC, NBS Program, and local hospitals in training healthcare professionals in medical and molecular genetics and developing new initiatives available for the Argentinean population.

Concerning genomics, the first genomes sequenced and the “100 Exome” campaign serve as a proof of concept, that it is possible to implement a local molecular diagnosis service based on next generation sequencing for research and clinical innovation. The implementation was based on the knowledge and capacity of local professionals. It is an example of how these projects can be set and then expanded in a national fashion, with guidelines and protocols adapted from the already existent internationally available services to satisfy the regional needs. Key to success of any of these techniques in the daily life of scientists and physicians is to create links between the different agents of change, to truly create interdisciplinary teams capable of tackling the problems that are sure to arise.

Next steps to strengthen the quality and availability of these genetic analyses include the creation of local human genetic databases of variant frequencies that truly represent the genetic imprint of the Argentinean population, which is underrepresented in international databases, with a questionable “Latino” ethnicity component, that fails to truly represent the Amerindian ancestry of Argentina and other Latin American countries.

In the public health and political field, new initiatives are required to expand the results achieved by the initial projects in the area of genomics. Some public pediatric hospitals, such as Hospital de Niños Dr. Ricardo Gutiérrez and Hospital Garrahan, have recently acquired some next generation sequencers and are already using them for diagnosing different pathologies. Moreover, several health insurance companies are starting to acknowledge the utility of these genomic tests and to cover the costs of diagnosis using these technologies. However, there is much to be done to expand the access to these tests to the whole population. Most advances are made in the central region of the country, while the other regions fall behind. In this case, it is imperative that policy makers of the country take into consideration this reality and promote the access and availability of these services in an ethical and egalitarian way.

## CONFLICT OF INTEREST

The authors state no conflict of interest.

## References

[mgg3455-bib-0001] Avena, S. , Via, M. , Ziv, E. , Pérez‐Stable, E. J. , Gignoux, C. R. , Dejean, C. , … Fejerman, L. (2012). Heterogeneity in genetic admixture across different regions of Argentina. PLoS ONE, 7(4), e34695 10.1371/journal.pone.0034695 22506044 PMC3323559

[mgg3455-bib-0002] Barbero, D. P. , Liascovich, R. , Rozental, S. , Botto, R. , Gramajo, S. , & Haefliger, C. (2003). Conocimientos de tocoginecólogos y pediatras acerca de la etiología y los factores de riesgo de los defectos congénitos. Archivos Argentinos de Pediatria, 101(3), 184.

[mgg3455-bib-0003] Bitgenia (2016). 100 Exomas. Retrieved from https://www.bitgenia.com/100exomas/files/bitgenia-100exomas-convocatoria.pdf

[mgg3455-bib-0004] Borrajo, G. J. C. (2007). Newborn screening in Latin America at the beginning of the 21st century. Journal of inherited metabolic disease, 30(4), 466–481. 10.1007/s10545-007-0669-9 17701285

[mgg3455-bib-0005] Borrajo, G. J. C. (2016). Newborn screening for phenylketonuria: Latin American consensus guidelines. Journal of Inborn Errors of Metabolism and Screening, 4, 1–5. 10.1177/2326409816682764

[mgg3455-bib-0006] Botto, L. D. , & Mastroiacovo, P. (2000). Surveillance for birth defects and genetic diseases. Genetics and public health in the 21st century, Oxford, UK: Oxford University Press.

[mgg3455-bib-0007] Botto, L. D. , Robert‐Gnansia, E. , Siffel, C. , Harris, J. , Borman, B. , & Mastroiacovo, P. (2006). Fostering international collaboration in birth defects research and prevention: A perspective from the International Clearinghouse for Birth Defects Surveillance and Research. American journal of public health, 96(5), 774–780. 10.2105/AJPH.2004.057760 16571708 PMC1470591

[mgg3455-bib-0008] Brenner, C. H. (2006). Some mathematical problems in the DNA identification of victims in the 2004 tsunami and similar mass fatalities. Forensic Science International, 157, 172–180. 10.1016/j.forsciint.2005.11.003 16361074

[mgg3455-bib-0009] Chiesa, A. , Prieto, L. , Mendez, V. , Papendieck, P. , de Luján Calcagno, M. , & Gruñeiro‐Papendieck, L. (2013). Prevalence and etiology of congenital hypothyroidism detected through an argentine neonatal screening program (1997‐2010). Hormone Research in Paediatrics, 80(3), 185–192. 10.1159/000354409 24008435

[mgg3455-bib-0010] Christianson, A. L. , Howson, C. , & Modell, B. (2006). Global report on birth defects: The hidden toll of dying and disabled children. White Plains, NY: March of Dimes Birth Defects Foundation.

[mgg3455-bib-0011] CODIS (1990). FBI. Combined DNA Index System Retrieved from https://www.fbi.gov/services/laboratory/biometric-analysis/codis

[mgg3455-bib-0012] CONADEP (Comisión Nacional sobre la Desaparicion de Personas) (1984). Nunca más, informe final de la Comisión Nacional sobre la Desaparición de Personas. Ed. Eudeba, Buenos Aires, Argentina.

[mgg3455-bib-0013] DEIS (Dirección de Estadísticas e Información de Salud, Ministerio de Salud) (2010). El Acceso a la Salud en Argentina: III Enceusta de Utilización y Gasto en Servicios de Salud. Retrieved from http://www.msal.gob.ar/fesp/images/stories/recursos-de-comunicacion/publicaciones/estudio_carga_enfermedad.pdf

[mgg3455-bib-0014] DEIS (Dirección de Estadísticas e Información de Salud, Ministerio de Salud) (2015a). Defunciones de Menores de Cinco Años Indicadores Seleccionados. Retrieved from http://www.deis.msal.gov.ar/wp-content/uploads/2016/12/Boletin156Menoresde5anos.pdf

[mgg3455-bib-0015] DEIS (Dirección de Estadísticas e Información de Salud, Ministerio de Salud) (2015b). Natalidad, mortalidad general, infantil y materna por lugar de residencia. Retrieved from http://www.deis.msal.gov.ar/wp-content/uploads/2016/12/BoletinNro154xlugardeResidencia.pdf

[mgg3455-bib-0016] DEIS (Dirección de Estadísticas e Información de Salud, Ministerio de Salud) (2016). Estadisticas Vitales: información Básica. Retrieved from http://www.deis.msal.gov.ar/index.php/tabulados-2/

[mgg3455-bib-0017] Di Lonardo, A. M. , Darlu, P. , Baur, M. , Orrego, C. , & King, M. C. (1984). Human genetics and human rights. Identifying the families of kidnapped children. The American Journal of Forensic Medicine and Pathology, 5(4), 339–347. 10.1097/00000433-198412000-00011 6441478

[mgg3455-bib-0018] Egeland, T. , Mostad, O. , Mevåg, B. , & Stenersen, M. (2000). Beyond traditional paternity and identification cases. Selecting the most probable pedigree. Forensic Science International, 110, 47–59. 10.1016/S0379-0738(00)00147-X 10802200

[mgg3455-bib-0019] Friedman, J. M. , Cornel, M. C. , Goldenberg, A. J. , Lister, K. J. , Sénécal, K. , Vears, D. F. , Global Alliance for Genomics and Health Regulatory and Ethics Working Group Paediatric Task Team (2017). Genomic newborn screening: Public health policy considerations and recommendations. BMC Medical Genomics, 10, 9 10.1186/s12920-017-0247-4 PMC5320805 28222731

[mgg3455-bib-0020] González‐Morón, D. , Vishnopolska, S. , Consalvo, D. , Medina, N. , Marti, M. , Córdoba, M. , … Kauffman, M. A. (2017). Germline and somatic mutations in cortical malformations: Molecular defects in Argentinean patients with neuronal migration disorders. PLoS ONE, 12(9), e0185103 10.1371/journal.pone.0185103 28953922 PMC5617183

[mgg3455-bib-0021] Groisman, B. , Bidondo, M. P. , Barbero, P. , Gili, J. A. , Liascovich, R. , Lopez Camelo, J. S. , & Grupo de Trabajo RENAC (2013). RENAC: national registry of congenital anomalies of Argentina.

[mgg3455-bib-0022] Groisman, B. , Bidondo, M. P. , Gili, J. A. , Barbero, P. , & Liascovich, R. (2013). Strategies to achieve sustainability and quality in birth defects registries: The experience of the National Registry of Congenital Anomalies of Argentina. Journal of Registry Management, 40(1), 29–31. 23778694

[mgg3455-bib-0023] Homburger, J. R. , Moreno‐Estrada, A. , Gignoux, C. R. , Nelson, D. , Sanchez, E. , Ortiz‐Tello, P. , … Bustamante, C. D. (2015). Genomic insights into the ancestry and demographic history of South America. PLOS Genetics, 11(12), e1005602 10.1371/journal.pgen.1005602 26636962 PMC4670080

[mgg3455-bib-0024] ICMP (2018). International Commission on Missing Persons. Retrieved from https://www.icmp.int/

[mgg3455-bib-0025] INDEC (Instituto Nacional de Estadística y Censos) (2010). Censo Nacional de Población, Hogares y Viviendas Retrieved from https://www.indec.gob.ar/

[mgg3455-bib-0026] Israel, M. (2013). Auge de consultas por la prueba genética que le hicieron a Angelina Jolie. Clarin. Retrieved from https://www.clarin.com/sociedad/auge-consultas-genetica-angelina-jolie_0_Hy2XFGOov7x.html

[mgg3455-bib-0027] Kling, D. , Egeland, T. , Piñero, M. H. , & Vigeland, M. D. (2017). Evaluating the statistical power of DNA‐based identification, exemplified by ‘The missing grandchildren of Argentina'. Forensic Science International: Genetics, 31, 57–66. 10.1016/j.fsigen.2017.08.006 28858673

[mgg3455-bib-0028] Kling, D. , Tillmar, A. O. , & Egeland, T. (2014). Familias 3‐Extensions and new functionality. Forensic Science International: Genetics, 13, 121–127. 10.1016/j.fsigen.2014.07.004 25113576

[mgg3455-bib-0029] Langou, G. D. , & Sachetti, F. C. (2017). Sustainable development goals and early childhood in Argentina: Gaps and priority actions to leave no one behind. Buenos Aires: CIPPEC.

[mgg3455-bib-0030] Lerman, G. (2017). Una pregunta. 30 años: Memoria escrita del Banco Nacional de Datos Genéticos. *Ministerio de Ciencia, Tecnología e Innovación Productiva*.

[mgg3455-bib-0031] Liascovich, R. , Rozental, S. , Barbero, P. , Alba, L. , & Ortiz, Z. (2006). A census of medical genetics services in Argentina. Revista Panamericana de Salud Pública, 19(2), 104–111. 10.1590/S1020-49892006000200005 16551384

[mgg3455-bib-0032] Ma, C. A. , Stinson, J. R. , Zhang, Y. , Abbott, J. K. , Weinreich, M. A. , Hauk, P. J. , … Milner, J. D. (2017). Germline hypomorphic CARD11 mutations in severe atopic disease. Nature Genetics, 49(8), 1192–1201. 10.1038/ng.3898 28628108 PMC5664152

[mgg3455-bib-0033] MSAL (Ministerio de Salud) (2011). Programa Nacional de Fortalecimiento de la Detección Precoz de Enfermedades Congénitas: Manual de Procedimiento. Argentina Retrieved from http://www.msal.gob.ar/images/stories/bes/graficos/0000000068cnt-p01-manual-de-procedimiento.pdf

[mgg3455-bib-0034] Nemirovsky, S. I. , Córdoba, M. , Zaiat, J. J. , Completa, S. P. , Vega, P. A. , González‐Morón, D. , … Kauffman, M. A. (2015). Whole genome sequencing reveals a de novo SHANK3 mutation in familial autism spectrum disorder. PLoS ONE, 10(2), e0116358 10.1371/journal.pone.0116358 25646853 PMC4315573

[mgg3455-bib-0035] Nosiglia, J. E. (1985). Botín de guerra. Cooperativa Tierra Fértil.

[mgg3455-bib-0036] Orrego, C. , & King, M. C. (1990). Determination of familial relationships. PCR Protocols: A guide to methods and applications, 4, (pp. 16–426). San Diego, CA: Academic Press.

[mgg3455-bib-0037] PAHO/WHO (Pan American Health Organization, World Health Organization) (2017). Core Indicator: Health Situation in the Americas.

[mgg3455-bib-0038] Penchaszadeh, V. B. (2013). Genetic testing and services in Argentina. Journal of Community Genetics, 4(3), 343–354. 10.1007/s12687-012-0093-1 22528519 PMC3739845

[mgg3455-bib-0039] PNUD (Programa de las Naciones Unidas para el Desarrollo) (2011). El sistema de salud argentino y su trayectoria de largo plazo: logros alcanzados y desafíos futuros. 1st edition. Buenos Aires

[mgg3455-bib-0040] Por los más de 40 años de lucha (2018). Pagina 12. Retrieved from https://www.pagina12.com.ar/116859-por-los-mas-de-40-anos-de-lucha

[mgg3455-bib-0041] RENAC (2017). Annual Report. *National Center of Medical Genetics, National Ministry of Health* Retrieved from http://www.anlis.gov.ar/cenagem/wp-content/uploads/2014/05/REPORTE-RENAC-2017-formato-web.pdf

[mgg3455-bib-0042] Ruitberg, C. M. , Reeder, D. J. , & Butler, J. M. (2001). STRBase: A short tandem repeat DNA database for the human identity testing community. Nucleic Acids Research, 29(1), 320–322. 10.1093/nar/29.1.320 11125125 PMC29767

[mgg3455-bib-0043] Seldin, M. F. , Tian, C. , Shigeta, R. , Scherbarth, H. R. , Silva, G. , Belmont, J. W. , … Alarcon‐Riquelme, M. E. (2007). Argentine population genetic structure: Large variance in Amerindian contribution. American Journal of Physical Anthropology, 132(3), 455–462. 10.1002/ajpa.20534 17177183 PMC3142769

[mgg3455-bib-0044] Stern, A. (2013). Science in the Service of Human Rights: Argentina 37 Years after the Coup. *HuffPost* Retrieved from https://www.huffingtonpost.com/alex-stern/argentina-dirty-war-dna_b_2941724.html

[mgg3455-bib-0045] Stevenson, R. E. , Hall, J. H. , & Goodman, R. M. (1993). Human malformation and related Anomalies. Oxford monographs on medical genetics N. 27, New York, NY: Oxford University Press.

[mgg3455-bib-0046] Therrell, B. L. , & Adams, J. (2007). Newborn screening in North America. Journal of inherited metabolic disease, 30(4), 447–465. 10.1007/s10545-007-0690-z 17643194

[mgg3455-bib-0047] Therrell, B. L. , Padilla, C. D. , Loeber, J. G. , Kneisser, I. , Saadallah, A. , Borrajo, G. J. , & Adams, J. (2015). Current status of newborn screening worldwide: 2015. Seminars in Perinatology, 39(3), 171–187. 10.1053/j.semperi.2015.03.002 25979780

[mgg3455-bib-0048] United Nations Data (UNData) (2017). Country Profiles, Argentina Retrieved from http://data.un.org/en/iso/ar.html

[mgg3455-bib-0049] Vázquez, M. , Rohr, C. , Brun, B. , Grisolia, M. , Méjico, G. , Gosso, M. F. , & Fay, F. (2017). Desarrollo del primer ensayo prenatal no invasivo (NIPT) en Argentina. Informe ALAC, XXII(1), 5–13.

